# Novel Biomarkers in the Diagnosis of Chronic Kidney Disease and the Prediction of Its Outcome

**DOI:** 10.3390/ijms18081702

**Published:** 2017-08-04

**Authors:** Jacek Rysz, Anna Gluba-Brzózka, Beata Franczyk, Zbigniew Jabłonowski, Aleksandra Ciałkowska-Rysz

**Affiliations:** 1 Department of Nephrology, Hypertension and Family Medicine, Medical University of Lodz, Zeromskiego 113, 90-549 Lodz, Poland; jacek.rysz@umed.lodz.pl (J.R.); bfranczyk-skora@wp.pl (B.F.); 2 Department of Nephrology, Hypertension and Family Medicine, WAM Teaching Hospital, Zeromskiego 113, 90-549 Lodz, Poland; 3 I Department of Urology, Medical University of Lodz, Zeromskiego 113, 90-549 Lodz, Poland; zb.jablonowski@gmail.com; 4 Palliative Medicine Unit, Chair of Oncology, Medical University of Lodz, Zeromskiego 113, 90-549 Lodz, Poland; olarysz@rmed.pl

**Keywords:** chronic kidney disease, biomarkers, ADMA, SDMA, uromodulin, KIM-1, NGAL, miRNA, ncRNA, lincRNA and proteomic and metabolomic biomarkers

## Abstract

In its early stages, symptoms of chronic kidney disease (CKD) are usually not apparent. Significant reduction of the kidney function is the first obvious sign of disease. If diagnosed early (stages 1 to 3), the progression of CKD can be altered and complications reduced. In stages 4 and 5 extensive kidney damage is observed, which usually results in end-stage renal failure. Currently, the diagnosis of CKD is made usually on the levels of blood urea and serum creatinine (sCr), however, sCr has been shown to be lacking high predictive value. Due to the development of genomics, epigenetics, transcriptomics, proteomics, and metabolomics, the introduction of novel techniques will allow for the identification of novel biomarkers in renal diseases. This review presents some new possible biomarkers in the diagnosis of CKD and in the prediction of outcome, including asymmetric dimethylarginine (ADMA), symmetric dimethylarginine (SDMA), uromodulin, kidney injury molecule-1 (KIM-1), neutrophil gelatinase-associated lipocalin (NGAL), miRNA, ncRNA, and lincRNA biomarkers and proteomic and metabolomic biomarkers. Complicated pathomechanisms of CKD development and progression require not a single marker but their combination in order to mirror all types of alterations occurring in the course of this disease. It seems that in the not so distant future, conventional markers may be exchanged for new ones, however, confirmation of their efficacy, sensitivity and specificity as well as the reduction of analysis costs are required.

## 1. Introduction

Characteristic features of chronic kidney disease (CKD) involve progressive destruction of the renal parenchyma and the loss of functional nephrons [1,2]. The loss of functional nephrons triggers molecular and cellular events responsible for compensatory growth of the remaining ones [3]. These mechanisms may become pathological and result in the development of renal lesions and lead to end-stage renal disease (ESRD) [4,5]. The development of chronic kidney disease involves the separation of podocytes from basal membrane and their loss with urine, therefore the determination of the presence of some structural proteins connected with glomerular barrier may be helpful in the diagnosis of renal diseases [6].

The loss of function in the course of chronic kidney disease is also associated with interstitial fibrosis and inflammation. Early diagnosis of this disease is important step in the prevention of CKD complications. Moreover, it is needed for the hampering of the progression to kidney failure and preventing the occurrence of cardiovascular events [7].

Currently, the diagnosis of CKD is made usually on the levels of blood urea and serum creatinine (sCr), however, sCr has been shown to be lacking high predictive value. Moreover, Steubl et al. [8] suggested that due to the curvilinear relationship between serum creatinine and estimated glomerular filtration rate (eGFR), creatinine serum concentrations were increased in serum only when approximately 40–50% of renal parenchyma was reversibly or irreversibly damaged. This may lead to the lack of detection of early stages of acute or chronic kidney failure and therefore to the delayed application of detailed diagnostics and the implementation of therapeutic interventions.

Reduced estimated glomerular filtration rate (eGFR), and increased urinary protein and albumin excretion as well as higher degrees of tubulo-interstitial atrophy and fibrosis are associated with poorer CKD prognosis [9]. These pathological alterations are preceded or stimulated by the infiltration by inflammatory cells, fibroblast activation and proliferation, excessive production and deposition of extracellular matrix components, and rarefaction of peritubular capillaries [9,10,11]. In early stages, symptoms of CKD are usually not apparent. Significant reduction of kidney function is the first obvious sign of disease. If diagnosed early (stages 1 to 3), the progression of CKD can be altered and complications reduced [12]. In stages 4 and 5 extensive kidney damage is observed which usually results in end-stage renal failure.

The unravelling of a part of molecular pathways associated with the aforementioned changes contributed to the understanding of renal disease [9,13]. There is a need for next generation biomarkers.Due to the development of genomics, epigenetics, transcriptomics, proteomics, and metabolomics, the introduction of novel techniques will allow for the identification of novel biomarkers in renal diseases [14].

Biomarker suitable for monitoring of CKD ought to have narrow biological variability in order to improve the assessment of longitudinal changes. Moreover, it should not be influenced by age, nutrition status or concurrent health concerns. A good biomarker should provide rapid, non-invasive and specific measurements correlating well with kidney tissue pathology [7]. Furthermore, good markers should be highly sensitive, specific for renal diseases, correlate with histopathological results of renal biopsy and disease progression, and enable the identification of early stages of renal impairment disease and prognosis [15].

Urine seems to be a better material for clinical diagnostics than blood because it can be collected non-invasively and it is relatively stable, probably due to long “storage” in the bladder. The collection of blood is inevitably associated with the activation of proteases and, consequently, with the generation of proteolytic breakdown products which are inevitably associated with its collection [16,17]. Currently, there are dipstick tests which changes colour in the presence of abnormalities such as excess amounts of protein, microalbuminuria, blood, pus, bacteria and sugar following the insertion into the urine sample. Urinalysis can be used to detect a numerous kidney and urinary tract disorders, such as chronic kidney disease, bladder infections and kidney stones [18].

Apart from traditional methods, saliva and exhaled breath are also tested as new, potentially useful sources of health information. The saliva urea nitrogen (SUN) dipstick has been suggested as a potential screening tool for acute and chronic kidney disease [19,20,21]. Raimann et al. [20] demonstrated that SUN strips have a higher screening sensitivity for detecting chronic kidney disease when applied alone but a better diagnostic specificity when used in combination with patient-reported urine output in patients with CKD stages 1–5/5 on dialysis. Moreover, another study revealed that increased SUN was an independent predictor of time to death in the Malawi population, which may suggest that SUN may serve as a biomarker of the patient’s disease severity and prognosis [21].

Also, exhaled breath was shown to contain chemicals having diagnostic value in human pathologies [22]. In vivo breath analysis of creatinine with the use of extractive electrospray ionization mass spectrometry (EESI-MS) was found to have high sensitivity and high specificity in patients with CKD.

The rapid development of high-throughput technologies and computer sciences as well as the application of systemic approaches resulted in impressive progression of personalized medicine. Currently, it seems that apart from disease diagnosis and treatment a great impact is made by predictive and preventative medicine and personalized health monitoring [23,24]. Systems biology in the field of personalized medicine focuses not only on disease mechanism explanations, but also on disease risk estimation, monitoring and disease prevention [23]. Genomic sequencing provides valuable information on disease risks and drug response efficiency. However, due to the fact that environmental factors may be also of key importance, genomic information may not always be sufficient [25]. According to studies, novel approaches such as transcriptomics, proteomics and metabolomics provide deeper insight into person’s phenotypes than genomic sequences alone, while the combination of genomic information together with aforementioned omics analysis delivers real-time information of a person’s physiological status [23]. Furthermore, it has been also suggested that apart from the analysis of genome, epigenome, transcriptome, proteome and metabolome, systems profiling of the gut microbiome, microRNA profiles and immune receptor repertoire may be important for health monitoring and personalized medicine. The gut microbiome has been recently considered as the “extended genome”, due to the fact that it may play an important role in drug metabolism [23,26].

This review presents some new possible biomarkers in the diagnosis of CKD and in the prediction of outcome.

## 2. Asymmetric Dimethylarginine (ADMA)

Asymmetric dimethylarginine (ADMA) is a novel biomolecule that can possibly serve as a biomarker in CKD. It is an analogue of L-arginine which naturally occurs in human circulation. It has been shown that increased levels of ADMA inhibit nitric oxide (NO) synthesis and therefore it impairs endothelial function stimulating renal impairment [27]. According to studies, high ADMA levels predicted a more accelerated course of renal function loss and promoted the development of renal damage due to the fact that it triggered glomerular hypertension, endothelial damage, salt accumulation, and cell senescence [27,28]. There are some possible molecular mechanisms of ADMA involvement in renal impairment. Matsuguma et al. [29] have suggested that elevated plasma concentration of ADMA is associated with levels of NG-dimethylarginine dimethylaminohydrolase (DDAH) protein which metabolizes ADMA and increased gene expression of enzyme protein methyltransferase (PRMT) which produces ADMA. Higher ADMA levels hamper the generation of endothelial nitric oxide and promote the development of hypertension in CKD. ADMA impairs endothelial function by diminishing arterial endothelial nitric oxide synthase (eNOS) phosphorylation via the inhibition of Ca/calmodulin-dependent protein kinase CaMKII. A decrease in eNOS phosphorylation is mediated by the mitogen-activated protein kinases (MAPK) pathway [30]. It can be reversed in vivo by increased catabolism of ADMA through dimethylarginine dimethylaminohydrolase-1 overexpression. Moreover, an in vitro study [31] demonstrated that the treatment with exogenous ADMA significantly increased stress fibre formation in human renal glomerular endothelial cells (HRGECs) and upregulated nuclear factor κB (NF-κB) and transforming growth factor β (TGF-β) expression.

ADMA is considered to be the “missing link” between cardiovascular disease and CKD [32]. According to studies, it is a strong biomarker predicting higher mortality in CKD [7,33,34,35] as well as a faster progression of kidney injury [36].

## 3. Symmetric Dimethylarginine (SDMA)

Symmetric dimethylarginine (SDMA) is a stable catabolic product of post-translationally methylated arginine-containing proteins which plays a vital role in basic cellular metabolism. SDMA is eliminated mainly by the kidneys [37]. Vallance et al. [36] observed higher concentrations of both SDMA and ADMA in haemodialysis patients. Serum and urine concentrations of SDMA have been shown to correlate with kidney dysfunction assessed on the basis of glomerular filtration rate (GFR) and creatinine clearance [38]. Kidney function deterioration was associated in that study with the increase in SDMA levels. Also, a large meta-analysis of 18 studies reported highly significant relationship between SDMA and kidney function [39]. According to studies, non-renal factors including muscle mass, diet, inflammation, diabetes, and oestrogen therapy had no significant impact on SDMA concentration [40]. Moreover, SDMA levels was demonstrated not to be influenced by acute inflammatory response, hepatic disease, cardiovascular disease or diabetes in the absence of concurrent kidney disease [41,42,43,44,45]. However, SDMA is affected, to a small extent, by obesity, gender, age, and polycystic ovary syndrome [40]. Another advantage of SDMA as a biomarker is its low intra-individual biological variability (5.8%) in comparison to cystatin C (8.6%) [46,47]. Kielstein et al. [48] demonstrated that SDMA was an early marker of change in GFR after living-related kidney donation.

## 4. Uromodulin

Uromodulin (also known as the Tamm–Horsfall protein) is a glycoprotein which according to studies is probably engaged in the protection of tubular cells from ascending urinary tract infections involved in chronic pyelonephritis and urolithiasis [49,50,51]. It is produced in the tubular cells of the thick ascending limb and the early distal tubule and released into the tubular lumen where it forms a layer on the tubular cell surface [52,53]. Uromodulin is highly abundant in urine [52]. It is also released in tubular cells into the interstitium, however, its physiological role there remains unknown [54]. Reduced urinary and serum concentrations of uromodulin are found in persons with interstitial fibrosis or tubular atrophy in the course of chronic kidney disease [55]. Uromodulin has been suggested as a promising biomarker for the number of intact nephrons, which indicates renal mass rather than kidney function. Uromodulin concentrations gradually decrease with worsening kidney function. In patients with CKD stage 2, levels are reported to be nearly 300 ng/mL, and drop below 10 in patients with CKD stage 5, while no uromodulin is detected in blood of anephric persons [51].The highest concentrations of uromodulin in persons without CKD were suggested to be due to the fact that no evasion mechanism for tubular function exists in contrary to glomerular filtration [8]. It has been suggested that plasma uromodulin could serve as a marker for kidney function in both CKD patients as well as those without CKD. In the study of Steubl et al. [8] the measurement of plasma concentrations of uromodulin enabled distinguishing between persons with CKD and patients at all stages of CKD with reasonable level of sensitivity and specificity. They provided evidence that uromodulin was a direct marker for the amount of intact tubular cells of the ascending limb and therefore it might be used as a marker for the number of remaining functional nephrons/renal tissue/tubular secretion [8].

Due to the fact that tubular function impairment associated with disturbed kidney function occurs early in the course of disease and it is mirrored by the decrease in uromodulin level, this glycoprotein can be used as an early biomarker. Moreover, Steubl et al. [8] observed a linear correlation with eGFR, while other markers (creatinine, blood urea nitrogen (BUN), and cystatin C) exhibit hyperbolic correlation to eGFR. Finally, uromodulin was shown to correlate significantly with proteinuria which is a strong predictor of CKD progression. Therefore, its role in the prediction of CKD progression was suggested, however, this requires the confirmation in further studies.

## 5. Kidney Injury Molecule-1 (KIM-1)

Kidney injury molecule-1 (T-cell immunoglobulin; mucin-containing molecule) is a type 1 trans-membrane protein [56]. KIM-1 has been shown to be up-regulated in dedifferentiated proximal tubule epithelial cells in kidney after ischemic or toxic injury [57,58]. It is not detected in healthy kidneys nor in urine. The increase in the amount of total protein in the glomerular filtrate and the presence of protein rollers that may increase glomerular pressure and contribute to the formation of ulceration of the tubules are thought to be direct factors inducing KIM-1. Up regulation of KIM-1 is a recognized consequence of proximal tubular damage in the nephron [56]. Moreover, it has been also found that the analysis of KIM-1 expression may be useful in the identification of glomerular injury [59]. Urinary KIM-1 has been shown to be a good predictor of renal injury prior to detectable changes in eGFR [60,61,62]. It was also suggested to be potential biomarker of chronic kidney disease due to tubulointerstitial damage [63]. Increased concentration of urinary KIM-1 may indicate the proximal tubular damage and it may reflect its involvement in phagocytosis of damaged proximal tubule epithelial cells by converting epithelial cells into semi-professional phagocytes [56,64,65]. The upregulation of KIM-1 was also suggested to be associated with the renewal of functional and morphological integrity of kidneys following ischemic insult [62]. Finally, the De Silva PMCS et al. [56] study showed that KIM-1 might be used to detect early CKD of uncertain aetiology in susceptible farming communities in Sri Lanka better than conventional markers.

## 6. Neutrophil Gelatinase-Associated Lipocalin (NGAL)

Neutrophil gelatinase-associated lipocalin (siderocalin, lipocalin-2 (LCN2) or lipocalin) is one of the first molecules triggering kidney development, converting embryonic mesenchymal cells into epithelial cells forming tubules and complete nephrons [66,67]. Leucocytes, loop of Henle and collecting ducts are the main sources of NGAL in the body [7,56]. It is expressed by tubular epithelial cells in response to injury and tubulointerstitial damage which frequently occur in the course of kidney disease progression [68]. The level of NGAL expression seems to be associated with the degree of kidney dysfunction and may help to indicate patients who are at higher risk of faster decline in kidney function. NGAL mediates the mitogenic effect of epidermal growth factor receptor (EGFR) signalling [2]. The activation of EGFR is associated with the stimulation of hypoxia-inducible factor (HIF-1α) and expression of LCN2 which results in enhanced cell proliferation, cytogenesis, renal damage, and CKD progression [2]. Urinary NGAL has been shown to be a good predictor of renal injury prior to detectable changes in eGFR [68,69]. A small study involving children with CKD stages 2–4 demonstrated that plasma NGAL levels inversely correlated with GFR and it outperformed cystatin C as a biomarker of kidney function in those with renal function below 30 mL/min [69]. Moreover, plasma and urinary NGAL was shown to have a predictive power for CKD progression even after adjustment for eGFR and reflected the severity of renal disease. It has been suggested that increased levels of NGAL might be associated with detectable damage occurring in loop of Henle and distal convoluted tubule [56].

## 7. miRNA, ncRNA, lncRNA and lincRNA Biomarkers

Epigenetic approaches towards the examination of the regulation of genes involved in disease detection and progression are now gaining wide interest. The discovery of the presence and sometimes a role of miRNA, ncRNA (non-coding RNA) and lincRNAs has led to attempts to use them as biomarkers.

**Figure 1:**
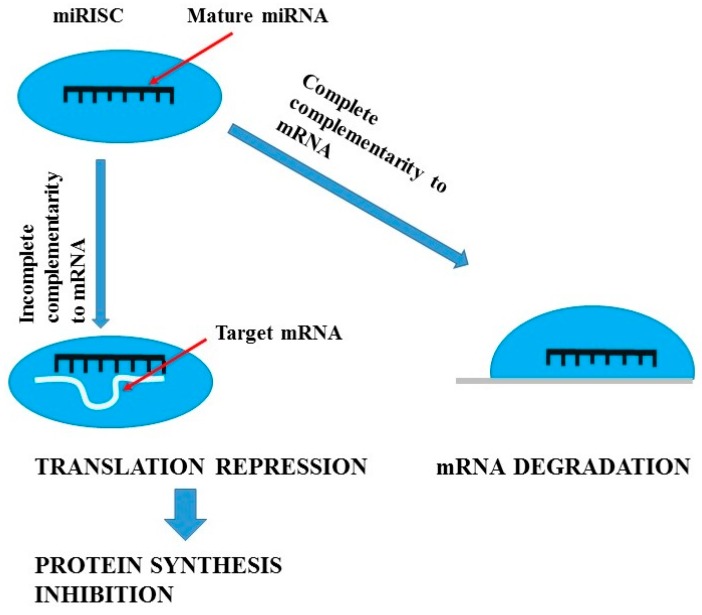
Mechanisms of miRNA action (figure was prepared and modified on the basis of [92]).

**Figure 2:**
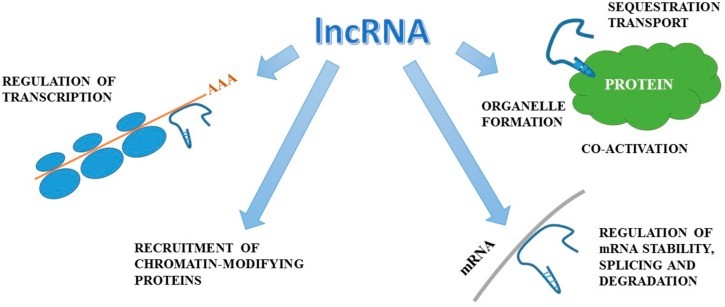
Mechanisms of action of lncRNA (figure was prepared and modified on the basis of [93]).The actions of lncRNA involve the interfering with protein-DNA binding, the regulation of mRNA stability and translation, the alteration of protein function, the organization of nuclear architecture and the modulation of mRNA levels and finally they are associated with the above mechanisms.

miRNAs are short (21–23 nucleotides long) endogenous antisense non-coding RNAs which act as post-translational repressors of gene expression [70,71,72]. Figure 1 presents mechanisms of miRNAs actions, while Figure 2 summarizes the actions of lncRNA. In kidneys they are involved in water homeostasis, osmoregulation, renin production, proximal tubule sodium and potassium handling, calcium sensing, renal development, and even renal senescence [72,73]. Moreover, miRNAs have been found to regulate kidney fibrosis through direct hampering or stimulation of matrix genes expression and through TGF-β signalling [74,75] and to modulate systemic and intra-renal inflammatory response [76,77]. miRNAs have been demonstrated to be involved in the progression of CKD and the development of diabetes, which is the most frequent cause of CKD. The Taïbi et al. [78] study of miRNA expression changes in the aortas of CKD and non-CKD wild-type mice and apolipoprotein E knock-out mice demonstrated that microRNAs may be implicated in endothelial and cardiovascular dysfunction in CKD. The results of other studies suggest that miRNAs play a key role in renal physiology. Global miRNA loss by selective elimination of Dicer in renal podocytes has been observed in severe glomerulopathies and tubular injury manifested by proteinuria and progressive renal functional impairment [79,80]. Studies performed on animal models have demonstrated that kidneys of Dicer [81] or microprocessor complex subunit DGRC8 (DiGeorge syndrome critical region gene 8) [82] knockout animals exhibit abnormal morphological development, glomerulocystic changes and renal failure. Due to the fact that miRNA processing in renal cells, especially in podocytes, is necessary for the maintenance of renal morphology and function and that podocytes play an important role in in diabetic and non-diabetic forms of CKD [83,84,85], it seems that miRNAs may prove highly useful and sensitive biomarkers of processes responsible for renal disease in humans. Studies on experimental models demonstrated that different tissue expression of specific miRNAs (miR-145,-143, -126, -223, -155, -125b) mirrored by their plasma levels may be used as non-invasive markers of vascular calcification and cardiovascular complications in CKD [78,86]. Moreover, replacement therapy with miR-145 was demonstrated to reduce atherosclerotic damage in experimental systems [87]. Diabetic kidney disease (DKD) and chronic allograft nephropathy (CAN) are frequent types of chronic renal impairment. In experimental models of diabetic kidney disease, miRNAs have been demonstrated to exert an impact on cell cycle progression, β cell dysfunction and insulin resistance enhancement [72]. Moreover, it has been revealed that miRNAs alter the transcription of inflammatory genes, stimulate extracellular matrix production, promote epithelial to mesenchymal transition, and enhance collagen production and fibronectin expression. DKD-associated miRNAs were shown to modify responses of mesangial, proximal tubular cells, and podocytes to transforming growth factor β (TGF-β) [88,89]. Numerous studies confirm the role of miRNAs in the pathogenesis and progression of DKD [77,90,91].

Renal transplantation improves survival and quality of life of ESRD patients and therefore it has become the treatment of choice [94]. However, according to estimates, long-term outcomes remain poor with ~50% of kidney transplant patients requiring dialysis 10 years after their surgery. Thus, there is a need for markers suitable for non-invasive monitoring of graft function for the presence of CAN. According to studies, the rate of renal function loss is associated with intra-renal miRNAs [95], and interstitial fibrosis with tubular atrophy (IF/TA) with urinary miRNAs. Three miRNAs (142-3p, 204, 211) have been suggested as specific biomarkers enabling the distinguishing between patients with CAN from those without with very high accuracy (area under the curve, AUC 0.96) [95,96,97]. However, their utility should be confirmed in further studies.

According to studies, circulating miRNAs usually tend to decline during severe chronic kidney disease and/or dialysis [86]. Circulating miR-21 has been shown to be associated with fibrosis score and to inversely correlate with estimated glomerular filtration rate in renal transplant recipients [98], while miR-29 and miR-200 families were diminished in urine exosomes of CKD patients with severe proteinuria [99]. It has been suggested that urinary miRNAs reflect the extent of laboratory manifestations, such as severity of proteinuria, eGFR, and histological severity of renal damage in numerous renal conditions, while circulating miRNAs seem to be positively associated with CKD severity [72]. Due to the fact that the kidney plays a key role in clearing small RNAs (including miRNAs) from the circulation, it seems that renal impairment is probably associated with large-scale changes in the circulating miRNAs as a result of their impaired clearance. Therefore, miRNAs may serve as impaired filtration markers which are better than conventional markers (including serum creatinine, cystatin-C and β2 microglobulin), but also as a sensitive biomarker of tubular dysfunction due to a miRNA transport mechanism in the proximal tubule [72]. MiRNAs fulfil many of the criteria for good biomarkers due to their stability in urine, sensitivity (rapid and significant release upon the development of pathology), specificity (able to differentiate pathologies of specific diseased organs), robustness (rapid and accurate detection by a number of technologies), and concentration-responsiveness to disease pathology as well as translatability between pre-clinical and clinical settings [72].

Khurana et al. [9] performed an analysis of non-coding RNA (ncRNA) from CKD patients and healthy control samples in order to identify biomarkers which will detect the presence of all stages of CKD. In their study 211 ncRNAs were found to show significant differences in their exosomal abundance (P-adj < 0.1) in CKD stage 1, 153 ncRNAs in stage 2, 221 ncRNAs in stage 3, and 117 ncRNAs in stage 4, compared to healthy controls. They suggested that the panel of 100 ncRNAs for which exosomal abundance differed considerably between patients with CKD 1 and 2 and healthy controls can be used as early markers for CKD. In turn, 67 overlapping ncRNAs were identified in patients with late stages of CKD. Moreover, Khurana et al. [9] found 27 differently abundant ncRNAs (P-adj < 0.1) in all the CKD stages, compared to healthy controls. Additionally, they identified 16 miRNAs out of which nine were significantly increased (let-7c-5p, miR-222–3p, miR-27a-3p, miR-27b-3p, miR-296-5p, miR-31-5p, miR-3687, miR-6769b-5p and miR-877-3p) and seven considerably decreased (miR-133a, miR-133b, miR-15a-5p, miR-181a-5p, miR-34a-5p, miR-181c-5p and miR1-2) in CKD patients in comparison to healthy controls. The most significant finding concerns miR-181a, for which exosomal abundance was about 200-fold lower in CKD patients than in healthy persons. The presence of miR-181a was earlier described in patients with nephrotic syndrome and those who underwent kidney transplantation. miR-181a has been suggested to be a potential biomarker used also in early diagnosis. Due to the fact that the biological role of differentially expressed urinary-derived exosomal miRNAs remains to be unravelled, it can only be hypothesized whether a significant increase of miR-181a in healthy controls is associated with enhanced export of miR-181a into exosomes from healthy kidney cells or whether miR-181a expression is down-regulated in kidney cells of CKD patients. Finally, Khurana et al. [9] reported eight antisense RNAs (EAF1-AS1, PCBP1-AS1, RP11-315I20.1, RP11-378E13.4, RP11-68I3.2, RP11-700F16.3, RP11-98D18.1 and RP11-1382.1) with different expression pattern in exosomes of CKD patients and healthy controls. These RNAs were either transcribed in the opposite orientation to introns of predicted protein-coding genes (7 RNAs) or transcribed in the opposite orientation to the *LIX1L* mRNA (1 RNA). According to studies, the role of long intergenic non-coding RNAs (lincRNAs) or antisense RNAs, which are transcribed opposite to the sense strand of mRNAs or sense to hnRNAs or primary transcripts, may involve the regulation of gene expression in eukaryotes. The diagnostic and prognostic utility of antisense RNAs is now analysed by numerous researchers in various diseases, especially in cancers [100,101,102]. Antisense lincRNAs may affect the expression of corresponding mRNA in cells. We are still beginning to understand the role of non-coding RNAs. Currently, the techniques used for their isolation and analyses (including Northern blotting, RT-qPCR, microarray, surface plasmon resonance and fluorescence-based techniques [103]), despite being technically advanced, do not provide results rapidly. Due to the fact that RNA-based sequences are quite small, their analysis frequently requires the conversion of transcripts into a pool of cDNAs constituting the sequencing library. Traditional identification of lncRNAs involves the creation of transcripts lists following the generation and sequencing of cDNA libraries or the probing of transcripts on tiling arrays [104]. The complicated methodology of isolation and analysis and the need for expensive equipment do not allow for the introduction of results into everyday clinical practise. However, we believe that in several years, the results of the most significant findings will be translated into diagnostic tests, perhaps in manner less simple than performing dipstick tests, but nonetheless less complicated and time- and effort-consuming than present methods. Smith et al. [103] made a step in this direction designing a method of electrochemical detection of miRNAs in a urine sample which requires minimal liquid handling in a straightforward dipstick-style test. Moreover, in their method urine sample can be small and there is no need for extensive miRNA extraction procedures as well as toxic and expensive reagents.

## 8. Proteomic and Metabolomic Biomarkers

As compared with currently available markers—serum creatinine and urinary albumin—proteomic biomarkers may facilitate more accurate and earlier detection of renal pathology [91]. Siwy et al. [105] in their study identified 287 disease-specific biomarkers for focal segmental glomerulosclerosis (FSGS), 291 for minimal change disease (MCD), 311 for membranous nephropathy (MN), 172 for lupus nephritis (LN), 509 for renal vasculitis, and 116 for IgA nephropathy (IgAN),as well as 619 diabetic nephropathy and nephrosclerosis (DN&N)-specific biomarkers. DN&N was associated with a reduction in haemoglobin level in comparison to the other types of CKD. Moreover, diminished small proline-rich protein 3 and leucine-rich repeat containing protein 25 and increased of clusterin and apolipoprotein fragments (with equal *p*-values) were found in this study. Siwy et al. [105] used urinary peptides to differentiate between distinct types of CKD. Many of the biomarker peptides found in their study were also indicated in other studies, which confirms the utility of some of these urinary peptides as specific biomarkers [16,106]. DN&N was distinguished from all other causes of CKD with an AUC of 0.92. The analysis of these proteins may help to avoid biopsies in cases where they are not needed. Proteomic signatures in MCD and FSGS are similar, in both cases reduced levels of fibrinogen are observed. MCD and FSGS are both primary podocytopathies in which a more severe injury leads to loss of podocytes in FSGS. Due to the fact that early in the clinical course renal biopsy may fail to detect glomerular scarring typical of FSGS, which results in incorrect diagnosis of MCD, novel diagnostic tools enabling the differentiation between MCD and FSGS would be highly welcome [107]. Siwy et al. [105] identified differences between MCD and FSGS in urinary peptides. They found that β-2-microglobulin was considerably down-regulated in MCD while α-1-antitrypsin was up-regulated in FSGS which is in agreement with other studies [108,109]. The differentiation between lupus nephritis and other types of CKD can be made on the basis of the calcium and zinc-binding protein S100-22 fragment, which is engaged in the regulation of inflammatory processes and the immune response. Increased serum levels of S100-21 protein were observed in systemic lupus erythematosus patients in comparison to healthy controls [110]. In a majority of CKD types, altered abundance of collagen fragments has been observed [105]. Pontillo et al. [111] suggested that urinary collagen fragments are early biomarkers for CKD (in patients with GFR > 60 mL/min/1.73 m^2^), however, they are not good markers in more advanced stages.

Apart from proteomics, metabolomics also provide more insight into disease mechanisms and therefore they have been utilized in the search for new biomarkers [112]. Recently, Good et al. [16] developed a CKD classifier based on 273 urinary peptides (CKD273) with high specificity and sensitivity for the diagnosis of CKD. This panel enabled more accurate discrimination between CKD patients and unaffected individuals than currently used markers (including albuminuria and serum creatinine) [16,113,114]. When the panel was analysed in a cohort of 34 healthy controls and 110 patients with CKD, 85.5% sensitivity and 100.0% specificity was obtained. Another examination of the specificity of identified markers after the inclusion of additional disease controls in the human urinary peptidome database yielded the overall specificity of 97.8% [16]. Due to the fact that most of the biomarker peptides in the urine are a product of proteolytic activity, disease-induced alterations in protease activities can be readily recognized through the analysis of proteolytic fragments [115]. Collagen fragments, especially fragments of collagen α-1 (I) chain, were found in the analysis of CKD273 panel to be the major constituents of urinary peptides [16]. These peptides, which probably mirror normal physiological turnover of the extracellular matrix [116], were demonstrated to be biomarkers suitable in the diagnosis of not CKD but also coronary artery disease (CAD) [16,117,118]. Nkuipou-Kenfack et al. [112] investigated the utility of proteomics and metabolomics in the assessment of renal function as well the performance of biomarkers in the prediction of renal function decline. They analysed urine samples using proteomics, and urine and plasma samples using metabolomics. As a result, they identified a panel of 30 metabolites (17 plasma and 13 urinary metabolites) which differed significantly between patients with early and with advanced stage CKD. At the same time, they identified a set of 46 peptides with significantly different distribution between groups. On the basis of potential biomarkers they designed three classifiers: plasma metabolite-based (MetaboP) urinary metabolite-based (MetaboU) and urinary peptide-based (Pept) and performed analyses. All of the aforementioned classifiers correlated very well with eGFR showing no significant differences between them [112]. The prognostic value of the classifiers was evaluated on the basis of the correlation with the follow-up data. The metabolite and peptide-based classifiers had good efficacy in the prediction of future renal function. Despite similar performance, the urinary peptide-based classifier was indicated to be slightly better than MetaboU and MetaboP (*p* = 0.1606 and 0.0879, respectively) [112]. The Nkuipou-Kenfack et al. [112] study demonstrated that the combination of urinary peptide, urinary metabolite and plasma metabolite biomarkers in a classifier (Pept_MetaboP+U) showed a good correlation performance with eGFR at baseline (*r* = 20.7833, *p* < 0.0001) and follow-up (*r* = 20.8061, *p* < 0.0001). However, due to the fact that the combination of classifiers was not better that their individual use, it seems that there is no need to perform combined proteomic and metabolomics analyses. Moreover, authors demonstrated reduced levels of most of collagen peptide fragments detected in urine samples, which was in agreement with other studies [55,119]. It seems that these changes may reflect alterations in the extracellular matrix (ECM) turnover and fibrosis which are common features of CKD [120,121]. CKD-related renal fibrosis is the endpoint of a cascade of events, including inflammation [122]. The presence of inflammatory process in CKD was in the Nkuipou-Kenfack et al. [123] study confirmed by the increased concentration of S100-22 protein which was responsible for stimulation of phagocytes migration. In turn, reduced urinary levels of uromodulin were in agreement with the observed interstitial fibrosis or tubular atrophy. The metabolomics sub-analysis revealed elevated levels of ADMA, hydroxykynurenine, and acylcarnitine in the plasma, and diminished ADMA in the urine [123]. According to Fouque et al. [124] accumulation of acylcarnitines in the plasma mirrors impaired clearance due to chronic kidney dysfunction. Acylcarnitines are the products of the esterification of acyl-CoA by l-carnitine. This estrification prevents the accumulation of acyl-CoAs generated in excess in renal failure [123,125], which may contribute to renal and cardiac lipotoxicity [126,127]. In turn, increased plasma levels of hydroxykynurenine have been shown to be associated with advanced stage CKD [128,129], however, the mechanisms of that relationship remains unclear. The significance of kynurenine metabolites in the progression of renal failure has been demonstrated in studies on arylformamidase-deficient mice. Arylformamidase catalyzes chemical reaction of *N*-formyl-l-kynurenine and water to formate and l-kynurenine. In Afmid-deficient mice, significantly higher plasma levels of kynurenine metabolites and the presence of renal failure were observed in comparison to wildtype mice. Moreover, glomerulosclerosis with increased mesangial matrix and decreased cellularity were revealed in kidney glomeruli of Afmid-deficient mice [130,131]. Korstanje et al. [132] found that the kynurenine 3-mono-oxygenase (*KMO*) gene (*Kmo*) was a candidate gene associated with albuminuria. In their study both knockdown of expression in zebrafish and genetic deletion of *Kmo* in mice resulted in proteinuria. The increase in kynurenine and kynurenic acid as well as a decrease in anthranilic acid found during the determination of tryptophan metabolites in the *Kmo*-KO mice suggested the occurrence of redirection of the pathway. Korstanje et al. [132] posed the hypothesis that tryptophan metabolism leading to NAD+ generation could be essential for podocytic microtubule formation and cytoskeleton rearrangements. Moreover, they suggested that due to the fact that NAD+ is also a cofactor for impairment of insulin sensitivity while insulin is crucial for podocyte function, diminished KMO activity in diabetes might contribute to the development of proteinuria in diabetic nephropathy [132,133,134].

## 9. Conclusions

Early diagnosis of CKD and identification of those likely to progress to end-stage renal disease (ESRD) has become highly important. Existing measures including creatinine level, estimated glomerular filtration rate (eGFR) and proteinuria seem to be insufficient. Therefore, new validated biomarkers are required for CKD progression and cardiovascular disease (CVD) risk. Complicated pathomechanisms of CKD development and progression require not a single marker but their combination in order to mirror all types of alterations occurring in the course of this disease. On the basis of aforementioned studies, it can be concluded that a panel of biomarkers rather a single marker is required to diagnose CKD with high sensitivity and specificity and to identify persons at high risk of progression. Moreover, it seems that in not so distant future, conventional markers may be exchanged for new ones, however, the confirmation of their efficacy, sensitivity and specificity as well as the reduction in analysis costs is required. The increasing number of studies concerning the search for new, sensitive and selective biomarkers useful for the diagnosis and quantitative assessment of mechanisms occurring in diseased kidneys confirms the importance of this issue.
